# Burden of hearing loss and diagnostic utility of a screening tool in Kazakhstan: insights from national and cross-sectional analyses

**DOI:** 10.3389/fpubh.2026.1788652

**Published:** 2026-05-12

**Authors:** Zhadra Bukenova, Galiya Orazova, Dinara Kassenova, Vafa Panahian, Aiman Mussina, Roza Suleimenova, Gulnur Zhakhina

**Affiliations:** 1 Department of Public Health and Hygiene, Astana Medical University, Astana, Kazakhstan; 2 Department of ORL, Astana Medical University, Astana, Kazakhstan; 3 Department of Ear, Nose and Throat Diseases, Azerbaijan Medical University, Baku, Azerbaijan; 4 Department of Public Health and Epidemiology, Astana Medical University, Astana, Kazakhstan; 5 Department of Medicine, Nazarbayev University School of Medicine, Astana, Kazakhstan

**Keywords:** audiometry, burden of disease, hearing loss, HHIA-S, sensitivity, specificity

## Abstract

**Background:**

Hearing loss is a significant public health concern that is often under recognized globally, with mild impairment accounting for the largest share of the burden. In Kazakhstan, little is known about national patterns of hearing loss or the utility of simple screening tools for detecting it. This study assessed the burden of hearing loss via Global Burden of Disease (GBD) data and evaluated the diagnostic performance of the Hearing Handicap Inventory for Adults–Screening version (HHIA-S) relative to audiometric testing.

**Methods:**

We analyzed GBD 2021 data to estimate the prevalence and years lived with disability (YLDs) from 1990 to 2021. Additionally, a cross-sectional survey was conducted among 506 adults recruited from primary healthcare facilities in Astana and nearby areas. All participants completed the HHIA-S questionnaire and underwent audiometric testing. Diagnostic accuracy was assessed via sensitivity, specificity, predictive values, and the area under the ROC curve (AUC).

**Results:**

In 2021, the age-standardized prevalence of hearing loss in Kazakhstan was 17,212 per 100,000 population (95% UI: 16,469-18,048), with mild hearing loss accounting for 71.9% of the cases. The YLD rate was 512 per 100,000 (95% UI: 346 s-732), showing stable trends since 1990. In the cross-sectional study, 20% of the participants had audiometrically confirmed hearing loss, with the prevalence rising sharply after the age of 60 years. Women composed 74% of the sample. The HHIA-S demonstrated a sensitivity of 70.2%, a specificity of 94.1%, a positive predictive value of 77.7%, a negative predictive value of 91.6%, and an AUC of 0.82, indicating good diagnostic performance.

**Conclusion:**

Hearing loss remains an important but under recognized health problem in Kazakhstan. Integrating simple screening tools such as the HHIA-S into primary care alongside strengthened audiological services and public awareness campaigns could facilitate early detection and reduce the long-term burden of untreated hearing impairment.

## Introduction

Hearing loss is a prevalent and often underdiagnosed public health concern with profound implications for communication, social engagement, and quality of life. It is often referred to as an invisible disability, yet it affects a substantial portion of the global population. According to estimates from the Global Burden of Disease (GBD) 2019 study, approximately 1.57 billion individuals [95% uncertainty interval (UI) 1.51–1.64], or 20.3% of the global population, lived with some degree of hearing loss in 2019 (1). Of these, 403.3 million [95% UI: 357.3–449.5] had moderate to higher severity hearing loss after adjusting for hearing aid use. The burden is expected to increase due to aging populations and increasing exposure to noise and other risk factors. Projections indicate that by 2050, this number will increase to 2.45 billion [95% UI, 2.35–2.56] (1). The economic impact of untreated hearing loss is substantial worldwide, amounting to nearly US$1 trillion each year (2).

Hearing loss refers to a reduction in hearing ability that can range from minimal perceptual disturbances to complete deafness (3). It may result from impairments in sound conduction to the inner ear, damage to the sensory cells of the cochlea, or disruptions in the auditory nerve pathways and cortical auditory centers that process sound. Hearing loss ranks as the third leading cause of years lived with disability (YLDs) across all ages worldwide, and it is the leading cause among individuals over 70 years of age (1). Among older adults, it is linked to increased risks of social isolation; cognitive decline, including dementia; falls; and various other adverse health outcomes (4, 5). Moreover, moderate to severe hearing loss poses challenges for both the youngest and the oldest age groups. Although hearing impairment is often overlooked, it constitutes a major public health issue. Moreover, in many low-and middle-income countries, including those in Central Asia, hearing loss remains insufficiently recognized within national health priorities (6). Comparative evidence from European countries shows that higher screening coverage and better access to hearing rehabilitation services are associated with earlier detection and reduced long-term burden, highlighting the critical role of health system capacity in shaping outcomes.

The World Health Organization (WHO) reported that more than half of all cases of hearing loss in adults can be prevented through primary prevention strategies (2). One of the leading preventable causes is exposure to excessive noise, while infections and the use of ototoxic medications also play significant roles. These health risks are particularly widespread in low- and middle-income countries, underscoring the need to prioritize prevention of hearing loss in such regions (7). Hearing impairment frequently results from a wide array of risk factors encountered throughout an individual’s life. Nevertheless, the specific contribution of each cause to the overall burden of hearing loss remains unclear (8). Moreover, structural factors such as urbanization, environmental noise exposure, and access to healthcare services may further influence the distribution and detection of hearing loss across populations.

In addition, contemporary public health frameworks emphasize that hearing loss is influenced not only by biological factors but also by broader social determinants of health, including socioeconomic status, access to healthcare, occupational exposures, and environmental conditions (9, 10). These perspectives align with established models of health inequalities aand burden of disease, which conceptualize health outcomes as the result of cumulative exposures and structural constraints across the life course. These factors may contribute to inequalities in both the risk of hearing loss and access to timely diagnosis and rehabilitation. According to the Hearing Care Framework, lower socioeconomic position increases unemployment, reduces aid uptake, and amplifies occupational risks, calling for equitable legislation and community-based care (11). Several studies advocate targeted policies for vulnerable groups, including expanded access in rural/low-income areas and noise protections, to mitigate inequalities in hearing loss prevention and rehabilitation (9, 10).

In Kazakhstan, approximately 150,000 individuals are officially registered as having hearing impairments (12); however, experts estimate that the actual number may be as high as 250,000, reflecting potential underdiagnosis and limited population coverage of screening programs (13). The highest prevalence of hearing loss is observed among adults aged 18 to 59 years, accounting for 20% of all reported cases (12), which underscores the growing public health relevance of hearing impairment among the working-age population. However, official statistical data on hearing loss in Kazakhstan remain scarce, with limited national reporting and no comprehensive population-based surveillance system. This lack of robust data hampers accurate assessment of the true burden, early detection efforts, and effective planning of audiological services and preventive interventions at the national level. Moreover, existing evidence from Kazakhstan is fragmented and does not integrate epidemiological burden estimates with the evaluation of practical screening approaches, highlighting a critical gap in both data availability and implementation research. This gap is not only geographical but also methodological, as the lack of integrated approaches combining epidemiological burden assessment with validation of accessible screening tools limits the development of scalable and effective public health strategies.

To improve the early identification of individuals with potential hearing problems, brief standardized tools such as the Hearing Handicap Inventory for Adults (HHIA) have been developed (8). Originally, it had 25 questions; however, there is a screening version that assesses the emotional and social impacts of hearing loss, and it is widely used for screening in both clinical and research settings (14). It offers a practical and reliable method to gauge the perceived handicap associated with hearing difficulties, particularly in large-scale population studies. However, the literature remains inconsistent regarding the effectiveness of different screening approaches. While some studies report high sensitivity and feasibility of self-reported tools in large-scale settings, others highlight limitations related to specificity, cultural adaptation, and variability across populations, underscoring the need for context-specific validation.

Therefore, the aims of this study were twofold: (1) to evaluate the burden of hearing loss in Kazakhstan using Global Burden of Disease data, and (2) to assess the effectiveness of the HHIA-S screening tool in identifying audiometrically confirmed hearing loss among adults. By comparing subjective assessments with objective audiometric data, we seek to determine whether such a tool could be feasibly implemented in primary care settings to facilitate earlier detection and intervention, ultimately reducing the burden of hearing loss in the country.

## Materials and methods

### Study design and population

To evaluate the burden of hearing loss, we analyzed publicly available data from the GBD, which is coordinated by the Institute for Health Metrics and Evaluation (15, 16). The GBD provides comprehensive, comparable estimates of disease burden across countries and over time. We extracted Kazakhstan-specific estimates of the prevalence, years lived with disability (YLDs), and age-standardized rates of hearing loss for the period from 1990–2021. These data were used to assess temporal trends and the evolving burden of hearing loss at the national level. All GBD data were accessed via the Global Health Data Exchange (GHDx).^1^

Additionally, to assess the effectiveness of screening tests in identifying hearing loss, a cross-sectional study was conducted between January and June of 2025 in the capital city and its nearby rural settlements. This study design was chosen as it allows for the evaluation of screening tool performance against a reference standard within a defined population at a single time point. Abovementioned areas represent both urban and rural populations with varying access to health services. The sample size was determined based on feasibility considerations and comparability with similar cross-sectional studies evaluating hearing screening tools; no formal *a priori* sample size calculation was performed.

### Participants of the study

Participants aged 18 years and older were recruited using a convenience sampling approach from individuals present in or near primary healthcare facilities in Astana, the capital of Kazakhstan, or in rural areas near Astana at the time of the study. This approach was chosen to ensure that participants could immediately undergo audiometric testing following the completion of the screening questionnaire, thereby minimizing loss to follow-up. Individuals with severe cognitive or communication impairments that prevented reliable self-reporting or audiometric testing were excluded. Two specially trained research staff members approached potential participants, explained the purpose of the study, and obtained informed consent. The participants then completed the electronic or paper-based HHIA screening survey on tablets. After completing the questionnaire, they were invited to a separate room for audiometric evaluation via a portable audiometer.

### Survey questionnaire

The survey consisted of two parts: (1) questions assessing the sociodemographic information of the participants and (2) the HHIA screening version. The questions concerned age, sex, living area (urban or rural), and self-reported hearing difficulties. The Hearing Handicap Inventory for Adults – Screening Version (HHIA-S) is a validated 10-item questionnaire (17, 18). The questionnaire was administered in Russian and Kazakh languages. The HHIA-S items were translated from English by bilingual researchers and compared with previously published Russian-language versions to ensure conceptual and linguistic accuracy (19, 20). The Kazakh version was back-translated and reviewed by native speakers to confirm semantic equivalence. A pilot test was conducted before the main survey to verify the clarity, cultural appropriateness, and linguistic consistency of the questions, and minor wording adjustments were made accordingly. The results of the pilot study have been published elsewhere (21).

The HHIA-S includes five items that reflect the emotional consequences of hearing loss and five that address social/situational difficulties. Each item is scored as “Yes” (4 points), “Sometimes” (2 points), or “No” (0 points), yielding a total score ranging from 0 to 40. A total score between 0 and 8 suggests a 13% likelihood of having hearing impairment. Scores ranging from 10 to 24 are associated with a 50% chance of hearing difficulties, whereas scores between 26 and 40 correspond to an estimated 84% probability of hearing impairment (22). In this study, a score of 10 or higher was considered indicative of hearing impairment. The HHIA-S has been shown to correlate well with audiometric hearing loss and is recommended for screening in community settings (23).

### Audiometric assessment

Pure-tone audiometry (PTA) is considered the gold standard in audiology diagnostics. In this study, PTA was performed using a clinical audiometer (Maico MA-28) equipped with AC headphones (DD65 v2) and bone-anchored headphones (B71 and B81) in a sound-isolated booth. Standard threshold audiometry involves the assessment of air-conduction thresholds at 250, 500, 1,000, 2000, 4,000, 6,000, and 8,000 Hz and bone-conduction thresholds at 500, 1000, 2000, and 4,000 Hz, with intensity adjusted in 5 dB increments.

The testing protocol used an automatic signal-interruption mode (500 ms tone followed by a 500 ms pause) (24). The hearing threshold at each frequency was defined as the lowest intensity level detected by the participant in at least 50% of the presentations. All assessments were conducted by a trained otorhinolaryngologist specializing in audiology. Before testing, all participants underwent otoscopic examination to rule out cerumen impaction and inflammation of the external or middle ear. According to the national protocol classification, hearing loss is defined as a hearing threshold of 25 dB or more in at least one better-hearing ear, which corresponds to grade 1 hearing loss (24).

### Statistical analysis

The burden of disease was quantified via age-standardized prevalence rates (ASPRs) and age-standardized years lived with disability (YLD) rates across the target region. YLDs were calculated using a prevalence-based method, consistent with approaches used by the WHO and Global Burden of Disease (GBD) studies. This method involves multiplying the number of prevalent cases by the corresponding disability weight (DW) for each condition (25). Age standardization was applied to eliminate confounding effects of differences in population structure, such as variations in age and sex distributions, enabling valid comparisons across regions. All rates are expressed per 100,000 individuals and presented with 95% uncertainty intervals (UIs). These intervals represent the estimated range of values, considering data variability and modeling assumptions, and are derived from 1,000 simulation runs, with the 2.5th and 97.5th percentiles indicating the bounds of the 95% UI. These data were analyzed to assess temporal trends in hearing loss burden in Kazakhstan by comparing age-standardized prevalence and YLD rates between 1990 and 2021.

For the cross-sectional study, descriptive analyses were used to summarize the characteristics of participants with and without audiometrically confirmed hearing loss. The binomial logistic regression models were constructed to assess associations between subjective hearing perception, HHIA-S scores, and audiometric outcomes, adjusting for potential confounders. Based on the HHIA-S score, a three-category variable was generated. A total score between 0 and 8 suggested a 13% likelihood of having hearing impairment. Scores ranging from 10 to 24 were associated with a 50% chance of hearing difficulties, whereas scores between 26 and 40 were associated with an estimated 84% probability of hearing impairment. For the multivariate logistic regression, variables were entered sequentially into the multivariable model. Multicollinearity was assessed using variance inflation factors. Model fit was evaluated using Hosmer–Lemeshow goodness-of-fit statistics.

To assess the diagnostic performance of the HHIA-S screening tool, pure-tone audiometry was used as the gold standard for calculating sensitivity, specificity, predictive values, and the ROC area. Receiver operating characteristic (ROC) curves were plotted, and the area under the curve (AUC) was calculated to evaluate the diagnostic performance of subjective measures as screening tools. The significance level was set at 0.05, and all the statistical analyses were conducted via Stata version 16.0.

## Results

### Trends in the prevalence and disability burden of hearing loss

Table 1 presents the age-standardized prevalence rates and years lived with disability (YLD) rates for overall hearing loss and its severity-specific subcategories in 1990 and 2021. In 1990, the total age-standardized prevalence of hearing loss was 17,201.56 per 100,000 [95% UI: 16,468.30–18,050.04], which remained relatively stable in 2021 at 17,212.58 [95% UI: 16,469.37–18,048.27]. Mild hearing loss constituted the majority of the burden in both years, with the prevalence increasing slightly from 12,321.21 to 12,370.63 per 100,000. In contrast, moderate, moderately severe, severe, and complete hearing loss all showed slightly lower prevalence.

**Table 1:** Trends in age-standardized prevalence and YLD rates of hearing loss by severity in Kazakhstan in 1990 and 2021.

Severity category	1990	2021	Trend
Age-standardized prevalence rates			
Hearing loss	17201.56 [16468.30–18050.04]	17212.58 [16469.37–18048.27]	↑
Mild hearing loss	12321.21 [11659.83–12991.62]	12370.63 [11704.49–13051.86]	↑
Moderate hearing loss	3430.99 [3021.18–3916.39]	3424.90 [3901.37–3003.52]	↓
Moderately severe hearing loss	869.94 [704.74–1064.80]	861.67 [771.48–1055.49]	↓
Severe hearing loss	235.46 [183.20–304.99]	223.57 [175.53–290.53]	↓
Profound hearing loss	236.11 [184.90–293.34]	236.19 [186.44–295.19]	↑
Complete hearing loss	107.85 [84.09–133.09]	95.44 [73.96–118.35]	↓
Age-standardized YLDs			
Hearing loss	517.27 [353.93–739.68]	512.62 [346.28–732.33]	↓
Mild hearing loss	168.41 [85.57–294.61]	169.22 [86.07–294.71]	↑
Moderate hearing loss	129.92 [79.66–197.96]	129.89 [79.65–194.39]	↓
Moderately severe hearing loss	91.90 [59.39–131.57]	91.25 [58.48–130.15]	↓
Severe hearing loss	44.05 [26.65–66.57]	41.99 [24.82–64.21]	↓
Profound hearing loss	55.55 [34.17–84.80]	55.90 [34.51–85.93]	↑
Complete hearing loss	27.44 [17.04–42.03]	24.38 [14.95–38.09]	↓

Age-standardized YLDs for total hearing loss also remained relatively stable, decreasing slightly from 517.27 [95% UI: 353.9–739.68] in 1990 to 512.62 [95% UI: 346.2–732.33] in 2021. Among the severity-specific categories, mild hearing loss accounted for the largest proportion of YLDs, and its burden slightly increased over time (from 168.41 to 169.22 per 100,000). Moderate and moderately severe hearing loss showed small reductions in YLDs, whereas profound hearing loss experienced a marginal increase from 55.55 to 55.90 per 100,000. Complete hearing loss and severe hearing loss showed minor declines in YLDs over the observed period.

### Screening test vs. audiometric diagnosis

This study included 506 participants. According to the screening questionnaire, 114 (22.5%) respondents had hearing loss, whereas only 103 (20%) had hearing loss on audiometric testing (Table 2). To assess the diagnostic performance of the HHIA-S screening tool, pure-tone audiometry was used as the gold standard. Table 2 presents the cross-classification of participants’ screening and audiometric results, along with calculated sensitivity, specificity, predictive values, and ROC area.

**Table 2:** Diagnostic performance of the HHIA-S screening questionnaire against pure-tone audiometry (gold standard).

A. Cross-tabulation (HHIA-S vs Pure-tone audiometry)			
	PTA Hearing loss	PTA Normal hearing	Total
Hearing loss	80	34	114
Normal hearing	23	369	392
Total	103	403	506
B. Diagnostic performance of HHIA-S (overall test performance)			
	Prevalence = 22.5% [19.0%–26.4%]		
	Sensitivity = 70.2% [60.9%–78.4%]		
	Specificity = 94.1% [91.3%–96.2%]		
	ROC area (AUC) = 0.82 [0.78–0.87]		
	Positive predictive value (PPV) = 77.7% [68.4%–85.3%]		
	Negative predictive value (NPV) = 91.6% [88.4%–94.1%]		

The screening test demonstrated a sensitivity of 70.2% (95% CI: 60.9–78.4) and a specificity of 94.1% (95% CI: 91.3–96.2). The positive predictive value (PPV) was 77.7% (95% CI: 68.4–85.3), whereas the negative predictive value (NPV) reached 91.6% (95% CI: 88.4–94.1). These findings indicate that the screening tool is highly effective in identifying individuals without hearing loss and performs well in detecting true cases of hearing impairment. The high NPV suggests that individuals who screen negative are unlikely to have clinically significant hearing loss as detected by pure tone audiometry. However, it should be noted that some individuals may still report hearing difficulties despite normal audiometric thresholds, reflecting limitations of pure tone testing in capturing functional hearing problems. As for PPV, although it is somewhat lower, indicating the presence of false positives, it remains within an acceptable range for population-level screening tools.

**Figure 1:**
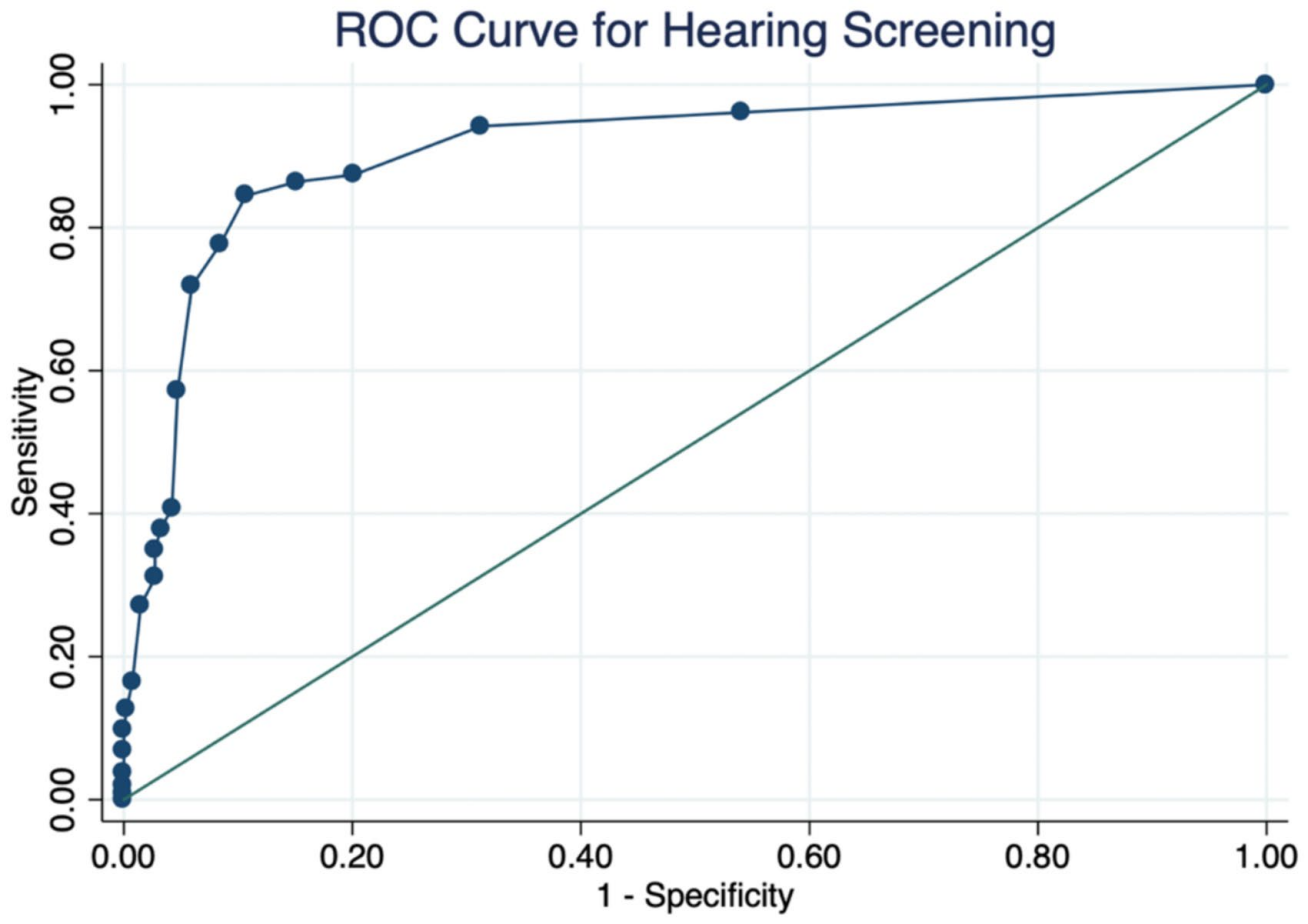
ROC curve for hearing screening.

The screening score, treated as a continuous variable, demonstrated excellent discriminative power, with an AUC of 0.91 (95% CI: 0.87–0.94) based on ROC analysis (Figure 1). However, when a predefined binary cutoff (score ≥ 10 indicating a positive screen) was used, the AUC decreased to 0.82 (95% CI: 0.78–0.87), reflecting the diagnostic performance at that specific threshold.

Additionally, the optimal threshold for the screening score was determined via the Youden index, which maximizes the sum of sensitivity and specificity. The cutoff value ≥10 yielded the highest Youden index (J = 0.74), with sensitivity of 84% and specificity of 89%.

### Characteristics of the participants and hearing impairment risk factors based on the audiometric test

Among the 506 participants, 132 (26%) were males, and 98 (19%) were retired (Table 3). In the cohort, 152 people answered the survey question “Do you have hearing problems?”; however, only 114 had test scores higher than 8. The mean age of the participants with hearing impairment was significantly greater than that of those without hearing impairment (53 ± 15 vs. 45 ± 15 years, *p* < 0.001). There was no significant difference in sex distribution between the groups: females accounted for 77 (75%) in the hearing-impaired group and 297 (74%) in the normal-hearing group (*p* = 0.827).

**Table 3:** Sociodemographic and psychosocial characteristics of the participants by audiometric hearing status (*n =* 506).

Characteristics	Total (*n =* 506)	Audiometry results		*p*-value
		Normal hearing (*n =* 403; 80%)	Hearing impairment (*n =* 103; 20%)	
Age, μ± SD	46 ( ± 16)	45 ( ± 15)	53 ( ± 15)	<0.001
Gender, *n* (%)				0.827
Female	374 (74)	297 (74)	77 (75)	
Male	132 (26)	106 (26)	26 (25)	
Living area, *n* (%)				0.008
Urban	337 (67)	257 (64)	80 (78)	
Rural	169 (33)	146 (36)	23 (22)	
Self-perceived hearing loss, *n* (%)				<0.001
Negative	354 (70)	326 (81)	28 (27)	
Positive	152 (30)	77 (19)	75 (73)	
Does a hearing problem cause you to feel embarrassed when meeting new people? *n* (%)				<0.001
No	337 (67)	294 (73)	43 (42)	
Sometimes	123 (24)	91 (23)	32 (31)	
Yes	46 (9)	18 (4)	28 (27)	
Does a hearing problem cause you to feel frustrated when talking to members of your family? *n* (%)				<0.001
No	357 (71)	329 (82)	28 (27)	
Sometimes	94 (18)	54 (13)	40 (39)	
Yes	55 (11)	20 (5)	35 (34)	
Do you have difficulty hearing when someone speaks in a whisper? *n* (%)				<0.001
No	318 (63)	298 (74)	20 (19)	
Sometimes	101 (20)	66 (16)	35 (34)	
Yes	87 (17)	39 (10)	48 (47)	
Do you feel handicapped by a hearing problem? *n* (%)				<0.001
No	424 (84)	375 (93)	49 (48)	
Sometimes	44 (9)	14 (3.5)	30 (29)	
Yes	38 (7)	14 (3.5)	24 (23)	
Does a hearing problem cause you difficulty when visiting friends, relatives, or neighbors? *n* (%)				<0.001
No	391 (77)	358 (89)	33 (32)	
Sometimes	68 (14)	27 (7)	41 (40)	
Yes	47 (9)	18 (4)	29 (28)	
Does a hearing problem cause you to attend religious services less often than you would like? *n* (%)				<0.001
No	448 (89)	381 (95)	67 (65)	
Sometimes	30 (6)	18 (4)	12 (12)	
Yes	28 (5)	4 (1)	24 (23)	
Does a hearing problem cause you to have arguments with family members? *n* (%)				<0.001
No	451 (89)	380 (94)	71 (69)	
Sometimes	42 (8)	21 (5.5)	21 (20)	
Yes	13 (3)	2 (0.5)	11 (11)	
Does a hearing problem cause you difficulty when listening to TV or radio? *n* (%)				<0.001
No	421 (83)	376 (93)	45 (44)	
Sometimes	46 (9)	15 (4)	31 (30)	
Yes	39 (8)	12 (3)	27 (26)	
Do you feel that any difficulty with your hearing limits or hampers your per-sonal or social life? *n* (%)				<0.001
No	407 (80)	370 (92)	37 (36)	
Sometimes	64 (13)	24 (6)	40 (39)	
Yes	35 (7)	9 (2)	26 (25)	
Do you feel that any difficulty with your hearing limits or hampers your per-sonal or social life? *n* (%)				<0.001
No	367 (72)	342 (85)	24 (23)	
Sometimes	85 (17)	47 (12)	38 (37)	
Yes	54 (11)	13 (3)	41 (40)	

A significantly higher proportion of participants with hearing impairment resided in urban areas compared with those with normal hearing (78% vs. 64%, *p* = 0.008). As expected, a greater proportion of individuals with audiometric hearing loss reported perceiving hearing problems (73%) than did those without hearing loss (19%) (*p* < 0.001).

In terms of communication difficulties and psychosocial experiences, individuals with audiometric hearing loss reported significantly more challenges across all 10 screening questions. For example, difficulties understanding others were reported by 47% of the hearing-impaired group, compared with only 10% of the normal-hearing group (*p* < 0.001). Similar trends were observed for problems watching TV or listening to the radio (26% vs. 3%), hearing in restaurants (40% vs. 3%), and experiencing frustration during conversations with family (4% vs. 5%) (all *p* < 0.001).

Table 4 presents the results of logistic regression analyses assessing factors associated with hearing impairment based on audiometric testing. Both unadjusted and adjusted models were examined to identify independent predictors. The *p*-values presented in Table 4 were obtained using the Chi-square test for categorical variables (or Fisher’s exact if data were not suitable for the Chi-square test), and the Mann–Whitney U test for continuous variables that were not normally distributed. In the unadjusted model, age was significantly associated with hearing impairment (OR = 1.03, 95% CI [1.02–1.05], *p* < 0.001), suggesting that each additional year of age increased the odds of hearing impairment by approximately 3%. However, after adjustment for covariates, this association was attenuated and no longer statistically significant. As for gender, it showed no significant association with hearing impairment in either model.

**Table 4:** Logistic regression analysis of factors associated with hearing impairment based on audiometric test.

Characteristics	Unadjusted model		Adjusted model	
	OR [95% CI]	*p*-value	OR [95% CI]	*p*-value
Age	1.03 [1.02–1.05]	<0.001	1.02 [0.99–1.05]	0.078
Gender (ref. female)	0.95 [0.58–1.55]	0.827	1.24 [0.59–2.58]	0.557
Living area (ref. rural)	1.98 [1.19–3.28]	0.008	2.88 [1.40–5.92]	0.004
Self perceived hearing loss (ref.no)	11.3 [6.88–18.7]	<0.001	3.21 [1.65–6.29]	0.001
HHIA-S category (ref. 13% likelihood of hearing impairment)				
50% chance of hearing difficulties	35.9 [18.8–68.6]	<0.001	25.9 [12.8–52.3]	<0.001
84% probability of hearing impairment	105 [38.1–289.4]	<0.001	43.8 [14.3–134.1]	<0.001

Living area demonstrated a significant effect. Individuals residing in urban areas (reference: rural) had higher odds of hearing impairment in the adjusted model (OR = 2.88, 95% CI [1.40–5.92], *p* = 0.004) compared with the unadjusted estimate (OR = 1.98, 95% CI [1.19–3.28], *p* = 0.008), suggesting that this variable retained its independent association after controlling for other factors.

Self-perceived hearing loss showed a strong and consistent relationship with audiometrically confirmed impairment. The odds were over 11 times higher in the unadjusted model (OR = 11.3, 95% CI [6.88–18.7], *p* < 0.001) and remained significantly elevated in the adjusted model (OR = 3.21, 95% CI [1.6–6.29], *p* = 0.001). Regarding HHIA-S category, participants with a 50% chance of hearing difficulties had 25.9-fold higher odds of audiometric hearing impairment after adjustment (OR = 25.9, 95% CI [12.8–52.3], p < 0.001). Those with an 84% probability of hearing impairment exhibited an even stronger association (adjusted OR = 43.8, 95% CI [14.3–134.1], p < 0.001).

## Discussion

This study provides a comprehensive overview of the burden of hearing loss in Kazakhstan, highlighting trends in prevalence and disability, as well as the performance of a hearing loss screening questionnaire in comparison with audiometric evaluation. These findings indicate that the age-standardized prevalence and disability burden (YLD) of hearing loss have remained relatively stable over the past three decades, with mild hearing loss accounting for the majority of the burden. In addition, based on our cross-sectional survey, we assessed the performance of a hearing screening tool and its correlation with audiometrically confirmed hearing loss. The screening tool demonstrated high specificity and strong negative predictive value, indicating its potential utility for identifying individuals unlikely to have hearing loss. Moreover, audiometric hearing loss was significantly associated with self-reported hearing and communication difficulties in noisy environments and during social interactions, underscoring the tool’s clinical relevance and the social impact of undiagnosed impairment.

The hearing loss burden in Kazakhstan aligns with the global patterns reported by the Global Burden of Disease (GBD) study, which also revealed that mild hearing loss constitutes the largest share of hearing impairment worldwide and that the overall burden has increased primarily because of population aging rather than increasing age-specific prevalence rates (1). These findings can also be interpreted within the Hearing Care Framework of social determinants of health, where hearing loss is influenced not only by biological aging but also by environmental exposures, access to healthcare services, and broader structural inequalities (11). Worldwide, the number of individuals living with hearing loss nearly doubled over the past three decades – rising from approximately 751.5 million in 1990 to 1.46 billion in 2019. During the same period, the age-standardized prevalence rate increased slightly, from 1,733.3 to 1,775.6 per 100,000 population (26).

Importantly, while age-standardized rates reflect risk adjusted for population age structure, the absolute number of cases may still increase due to demographic growth and population aging. The relative stability in the age-standardized prevalence and YLDs of hearing loss in Kazakhstan reflects global patterns, where the overall increase in hearing loss burden is primarily attributed to population aging rather than rising age-specific prevalence. Despite the nearly twofold global increase in the number of individuals affected, the age-standardized prevalence rate has shown only a modest rise, suggesting that improvements in healthcare access and early detection may have mitigated sharper increases in age-adjusted rates.

For the subgroup analysis by hearing loss type, the decline in the prevalence of more severe forms of hearing loss (moderate, moderately severe, and severe) may reflect improved access to healthcare services, earlier detection (27), and possibly the impact of public health measures aimed at reducing ototoxic exposure or untreated infections. In addition, between 2011 and 2019, Kazakhstan implemented two State Healthcare Development Programs that placed strong emphasis on public health education and promotion efforts (28, 29). These initiatives may have contributed to increased public awareness of hearing loss and supported enhancements in how hearing impairments are identified and managed within the healthcare system. However, the slight increase in the burden of profound hearing loss suggests that challenges remain, particularly in ensuring timely intervention for individuals at the extreme end of the severity spectrum.

This study also demonstrated that the HHIA-S screening questionnaire, while not a diagnostic instrument, exhibited strong screening performance, characterized by high specificity and a robust negative predictive value. Similar findings were reported in a population-based study conducted in Brazil, where the HHIA-S screening questionnaire exhibited a specificity of 75% and a sensitivity of 47%, indicating its better performance in correctly identifying individuals without hearing loss than detecting those with the condition (30). In contrast, another cross-sectional study demonstrated more favorable diagnostic characteristics of the HHIA-S, with a sensitivity of 89.1% and a specificity of 75.0% (31), suggesting that under certain conditions or in specific populations, the tool can achieve both high sensitivity and specificity.

The findings of the current study support its utility as a population-level screening method in resource-constrained settings, where audiometric testing may not be feasible for all individuals. The sensitivity of the HHIA-S was 70.2%, indicating that one-third of hearing loss in this population is not detected by the screening tool alone. While this level of sensitivity is acceptable for a community-based screening tool, it highlights the potential for false-negative results. In practice, screening questionnaires such as the HHIA-S should be considered first-stage screening instruments rather than diagnostic tools. A high AUC in the results indicates strong overall discriminative capacity (32), consistent with prior studies that used self-reported hearing difficulty as a proxy for audiometrically confirmed hearing loss (33, 34). Nevertheless, the lower PPV and some false positives suggest that while the tool is valuable for ruling out hearing loss, confirmatory testing remains necessary for definitive diagnosis. The findings of this study highlight the practical relevance of combining subjective and objective measures in screening strategies, particularly in settings where access to formal audiometric testing is limited.

The sociodemographic and psychosocial correlates of hearing loss identified in our study also merit attention. Older age, urban residence, and self-reported hearing problems were independently associated with audiometric hearing loss, consistent with the global literature, which has shown that age-related hearing loss is the most common form of hearing impairment (35, 36). Interestingly, urban residence emerged as a risk factor, potentially reflecting better access to diagnostic services or greater exposure to occupational and environmental noise or stress-related health effects. This observation may also reflect underlying health inequalities, including differential access to diagnostic services and varying exposure to environmental and occupational risk factors. Therefore, the relationship between urban living and hearing loss should be interpreted with caution. Urban living may be a marker of exposure to an unknown environmental or occupational factor, such as street traffic and machinery noise (37, 38). Unfortunately, we do not have sufficient information on this. Therefore, there is a high possibility of uncontrolled confounding variables, and further studies are needed.

The results of this study revealed strong associations between objective hearing impairment and self-reported communication difficulties, particularly in social settings such as restaurants, cinemas, and family conversations. These findings are aligned with studies that underscore the psychosocial impact of hearing loss, including social withdrawal, reduced quality of life, and increased risk of depression and cognitive decline (39, 40). For example, participants who reported frustration during conversations or difficulty hearing in public venues were significantly more likely to have audiometrically confirmed hearing loss, highlighting the validity of these subjective experiences as important clinical indicators.

These results emphasize the importance of integrating psychosocial dimensions into screening and management strategies. While some screening tools focus solely on auditory function, a more holistic approach that incorporates perceived hearing difficulties and their impact on daily functioning may enhance the identification of individuals most in need of intervention (41, 42).

Our findings also support the broader call by the World Health Organization (WHO) for population-based hearing screening programs and improved access to hearing aids and rehabilitative services, especially in low- and middle-income countries where the burden of unaddressed hearing loss remains disproportionately high (2). Despite the known benefits of early diagnosis and intervention, including improved communication, social engagement, and mental health, barriers such as stigma (43), lack of awareness (44), and limited audiological infrastructure continue to hinder progress. Similar barriers have been reported in both high-income and low- and middle-income settings, although their magnitude varies depending on health system capacity and access to audiological services, highlighting the importance of context-specific approaches. These findings can be interpreted within the framework of social determinants of health and health inequality models, which emphasize that access to diagnosis and rehabilitation is shaped by structural, socioeconomic, and health system factors. From a policy perspective, our results support the feasibility of integrating low-cost, questionnaire-based screening tools into routine primary care, particularly in resource-constrained settings.

Against this background, the results of our study gain particular relevance in Kazakhstan, where recent reforms in the provision of care for patients with hearing impairment are currently underway. In September 2025, legislative amendments were adopted introducing a more functional approach to the determination of hearing impairment. According to the new regulations, individuals with clinically confirmed hearing loss are eligible to receive state-funded hearing aids. Importantly, this applies even in the absence of a formally established disability status. This step reflects a gradual shift in national policy toward expanding access to hearing rehabilitation and aligns with international recommendations aimed at reducing the burden of untreated hearing loss. However, the effective implementation of these measures faces several systemic challenges. Currently, the country lacks a national register of patients with hearing impairments, which complicates accurate estimation of disease prevalence, monitoring of hearing aid needs, and healthcare resource planning. Furthermore, the absence of standardized patient pathways between levels of care may lead to delays in diagnosis and referral to specialists. In this context, the introduction of simple screening tools, such as the HHIA-S, into routine primary healthcare practice could represent an important first step toward building a more structured system for identifying hearing impairments. This brief questionnaire could be used during preventive check-ups, chronic disease visits, or geriatric consultations. As it does not require specialized equipment and requires minimal time, it can be administered by general practitioners and trained nurses, making it particularly suitable for resource-limited settings and areas with a shortage of audiological specialists. An additional direction involves the implementation of community-level screening programs to expand population coverage, particularly among older adults and residents of rural or remote areas. Such initiatives could be delivered through mobile medical teams, public health campaigns, or integration with screening programs for other chronic diseases. These approaches may help reduce geographical barriers to healthcare access and improve public awareness of hearing health.

Early detection through systematic screening is critical, as hearing loss is often diagnosed at advanced stages when communication difficulties already significantly affect quality of life, social functioning, and mental wellbeing. Earlier identification can improve rehabilitation outcomes and enhance the effectiveness of recently expanded hearing aid programs.

Additionally, the implementation of structured screening at the primary healthcare level could serve as a foundation for establishing a national monitoring system for hearing impairments. In the long term, this may include the development of an electronic patient registry integrated with existing digital health systems, as well as the establishment of standardized referral pathways across levels of care. Such measures would improve case detection, enhance access to rehabilitative services, and support more efficient healthcare resource allocation.

Beyond its practical implications, this study contributes to the literature by providing an integrated perspective that combines epidemiological burden assessment with the evaluation of a scalable screening tool within a real-world healthcare context.

Future research should incorporate longitudinal designs to assess the progression of hearing loss over time and evaluate the long-term effectiveness of screening interventions. In addition, studies integrating socioeconomic, environmental, and health system variables are needed to better understand structural determinants and inform targeted public health strategies.

In the long term, the development of these mechanisms could substantially strengthen the healthcare system’s capacity to respond to the growing burden of hearing loss, particularly in the context of population aging and increasing demand for rehabilitative services.

### Strengths and limitations

This study provides the first comprehensive assessment of hearing loss in Kazakhstan, combining Global Burden of Disease data with original population-based survey results. A key strength lies in its dual design: the use of GBD estimates allowed for long-term trend analysis, whereas the cross-sectional survey provided novel insights into the performance of the HHIA-S screening tool in real-world settings. The study also benefited from standardized audiometric testing performed by trained specialists, ensuring high-quality objective measurements.

At the same time, several limitations must be acknowledged. The cross-sectional survey included a relatively small sample size (*n =* 506) and was geographically limited to Astana and nearby areas, which may affect its representativeness at the national level. Moreover, no formal *a priori* sample size calculation was done. The sample size was determined primarily on feasibility grounds and to ensure comparability with similar cross-sectional studies evaluating hearing screening tools. As a result, the statistical power to detect certain associations may be limited, and the findings should be interpreted with appropriate caution. Future studies with larger, systematically determined sample sizes would strengthen the robustness and generalizability of the results. Recruitment through primary healthcare facilities may have introduced selection bias, underrepresenting individuals who do not seek medical care, especially in remote rural areas. In addition, convenience sampling reduces the external validity of findings. The sample was also skewed toward women (74%), potentially influencing prevalence estimates and associations with risk factors. The cross-sectional design limits the ability to establish causal relationships between variables. Furthermore, important sociodemographic covariates, such as education, income, and occupation, were not collected, which may have provided additional explanatory power. Due to the study design and available data, formal robustness or sensitivity analyses could not be performed; therefore, the findings should be interpreted with caution and may not be directly generalizable to the national population. Future studies with larger, more representative samples are needed to confirm these results.

Another limitation relates to the self-reported HHIA-S screening tool: although validated, it relies on subjective perception, which can be influenced by mood, cognitive state, or stress. Future studies may benefit from combining questionnaires with simple clinical methods such as the whisper test to improve sensitivity. Additionally, while urban residence emerged as a significant risk factor, the study did not directly measure environmental noise, occupational exposure, or other ecological determinants, restricting the interpretation of this finding.

Despite these limitations, this study provides valuable evidence for improving hearing screening strategies in Kazakhstan and highlights the need for larger, more representative, and longitudinal studies to strengthen the evidence base for public health interventions.

## Conclusion

Hearing loss remains a significant but underrecognized public health issue in Kazakhstan, with mild forms constituting the largest proportion of the burden. The long-term trends in age-standardized prevalence and YLDs have remained relatively stable, reflecting global patterns driven primarily by demographic aging. The cross-sectional component of the study demonstrated that the HHIA-S screening tool offers high specificity and reasonable diagnostic utility, particularly in ruling out hearing loss, although its sensitivity remains moderate. These results underscore the importance of integrating simple and accessible screening tools such as the HHIA-S into primary care settings, especially in rural or underserved areas, to facilitate early identification and referral. To address this growing need, Kazakhstan should prioritize the expansion of community-based screening initiatives, improve public awareness, and strengthen audiological services across all levels of the health system.

## Data Availability

The original contributions presented in the study are included in the article/supplementary material, further inquiries can be directed to the corresponding author.

## Footnotes

1
http://ghdx.healthdata.org/gbd-results-tool
